# A robust and efficient method for the extraction of plant extracellular surface lipids as applied to the analysis of silks and seedling leaves of maize

**DOI:** 10.1371/journal.pone.0180850

**Published:** 2017-07-11

**Authors:** Derek M. Loneman, Layton Peddicord, Amani Al-Rashid, Basil J. Nikolau, Nick Lauter, Marna D. Yandeau-Nelson

**Affiliations:** 1 Department of Genetics, Development & Cell Biology, Ames, Iowa, United States of America; 2 NSF-Engineering Research Center for Biorenewable Chemicals, Iowa State University, Ames, Iowa, United States of America; 3 Department of Plant Pathology and Microbiology, Iowa State University, Ames, Iowa, United States of America; 4 Interdepartmental Genetics & Genomics Graduate Program, Iowa State University, Ames, Iowa, United States of America; 5 Young Engineers and Scientists Research Program for high school students, Iowa State University, Ames, Iowa, United States of America; 6 Roy J. Carver Department of Biochemistry, Biophysics & Molecular Biology, Iowa State University, Ames, Iowa, United States of America; 7 Center for Metabolic Biology, Iowa State University, Ames, Iowa, United States of America; 8 USDA-ARS, Corn Insects and Crop Genetics Research Unit, Iowa State University, Ames, Iowa, United States of America; Universidade do Porto, Faculdade de Farmácia, PORTUGAL

## Abstract

Aerial plant organs possess a diverse array of extracellular surface lipids, including both non-polar and amphipathic constituents that collectively provide a primary line of defense against environmental stressors. Extracellular surface lipids on the stigmatic silks of maize are composed primarily of saturated and unsaturated linear hydrocarbons, as well as fatty acids, and aldehydes. To efficiently extract lipids of differing polarities from maize silks, five solvent systems (hexanes; hexanes:diethyl ether (95:5); hexanes:diethyl ether (90:10); chloroform:hexanes (1:1) and chloroform) were tested by immersing fresh silks in solvent for different extraction times. Surface lipid recovery and the relative composition of individual constituents were impacted to varying degrees depending on solvent choice and duration of extraction. Analyses were performed using both silks and leaves to demonstrate the utility of the solvent- and time-optimized protocol in comparison to extraction with the commonly used chloroform solvent. Overall, the preferred solvent system was identified as hexanes:diethyl ether (90:10), based on its effectiveness in extracting surface hydrocarbons and fatty acids as well as its reduced propensity to extract presumed internal fatty acids. Metabolite profiling of wildtype and *glossy1* seedlings, which are impaired in surface lipid biosynthesis, demonstrated the ability of the preferred solvent to extract extracellular surface lipids rich in amphipathic compounds (aldehydes and alcohols). In addition to the expected deficiencies in dotriacontanal and dotriacontan-1-ol for *gl1* seedlings, an unexpected increase in fatty acid recovery was observed in *gl1* seedlings extracted in chloroform, suggesting that chloroform extracts lipids from internal tissues of *gl1* seedlings. This highlights the importance of extraction method when evaluating mutants that have altered cuticular lipid compositions. Finally, metabolite profiling of silks from maize inbreds B73 and Mo17, exposed to different environments and harvested at different ages, revealed differences in hydrocarbon and fatty acid composition, demonstrating the dynamic nature of surface lipid accumulation on silks.

## Introduction

The plant cuticle is the outermost chemical barrier between aerial portions of a plant and the external environment. The cuticle is a structure deposited by epidermal cells and consists of an insoluble polyester, cutin, which is embedded and coated with a complex chemical mixture of extracellular, non-polar and amphipathic lipids that are derived from fatty acid precursors [1], and can include long-chain fatty acids, primary and secondary alcohols, aldehydes, wax esters, ketones and linear hydrocarbons. These lipids are sometimes referred to as “cuticular and epicuticular waxes” and are referred to herein as “extracellular surface lipids”, to avoid confusion with waxes, which are normally very-long chain esters. Collectively, the hydrophobic nature of these surface lipids confers a functional role as a water barrier between the organism and its environment, and is thus important in modulating water status [1, 2]. The concentration and composition of the extracellular surface lipid metabolome has been shown to vary widely across organisms [3–5], as well as among organs and tissues or across stages of development within an individual organism [3, 6].

To facilitate quantitative genetic and applied breeding efforts that target crop improvement via enhanced tolerance to environmental stressors mediated by the cuticle (e.g., water status regulation, frost resistance, plant-pest and -pathogen interactions, and protection from UV irradiation [1]), we require methods that simultaneously extract both hydrophobic and amphipathic extracellular surface lipids, and that can be efficiently applied to large-scale experiments. Recent optimization of lipid extraction methods for large numbers of samples has been a particular focus with microalgae [7–11], as related to the biological engineering of molecules that have biofuel applications. These methods are primarily directed at the medium- to high-throughput extraction of total cellular lipids, and are improvements on the lower-throughput, classical methods for extraction of homogenized animal tissues published by Folch [12] and Bligh and Dyer [13], which differed in the amounts of solvent and the relative ratios of the chloroform-methanol extraction solution. Efficient extraction of extracellular surface lipids in plants, however, requires a rapid and efficient method for non-homogenized tissue that recovers both amphipathic and hydrophobic lipid classes that frequently accumulate on plant surfaces, while limiting the extraction of internal lipids. A commonly-used method for epicuticular and intracuticular lipid extraction comprises submerging fresh tissue in a solvent of moderate polarity [4, 14, 15] commonly for 30 s to 1 min, which dissolves the extracellular surface lipids and allows for subsequent analysis via gas chromatography-mass spectrometry (GC-MS). Chloroform is frequently used as an extraction solvent, based on its ability to support high and reproducible lipid yields in numerous plant species [16], and has been shown as an effective solvent for lipid extraction from fruit and leaf surfaces of several crop species (tomato, apple and aspen) [17]. Alternatively, hexanes have been utilized as an extraction solvent for cuticular waxes of leaves and stems from Arabidopsis [18], and for hydrocarbon extraction from silks of maize.

Maize silks, which are the stigmatic portions of the female flowers and are exposed to environmental stresses during the critical period of pollination, possess a unique composition of surface lipids that is rich in simple long-chain hydrocarbons (e.g. n-alkanes, n-monoenes and n-dienes), and contains smaller amounts of long chain fatty acids and aldehydes [19, 20]. Previous profiling of extracellular surface lipids on maize silks was based on extractions with either chloroform [19, 20] or hexanes [20, 21]. While hexanes seem ideal as a non-polar solvent for extraction of hydrocarbons, it remains unclear what the most efficient and selective solvent is for the extraction of tissues rich in both non-polar and amphipathic surface lipids, as is observed on maize silks. Moreover, a potential limitation of each of the commonly used methods for extraction of extracellular surface lipids is the short extraction time, often only 30 s to 1 min, which requires that each sample be extracted sequentially. Development of an extraction method that allows for simultaneous or staggered extraction of large numbers of samples would be useful when conducting large-scale experiments.

The aims of this study were 1) to develop an efficient and selective extraction method for use in large-scale experiments that simultaneously extracted non-polar (i.e. hydrocarbons) and amphipathic (i.e. fatty acids, aldehydes) extracellular surface lipid metabolites from maize silks; 2) to demonstrate the effectiveness of this extraction method on maize leaf tissue, using leaves from wildtype and *glossy1* maize seedlings, which are defective in accumulation of surface aldehydes and alcohols; and 3) to apply the method to characterize changes in the silk extracellular surface lipid metabolome as the silks emerge from the encasing husk leaves into the external environment.

## Materials and methods

### Plant materials and sample processing

#### Maize silk experiments

Maize (*Zea mays* L.) inbreds B73 (PI 550473) and Mo17 (PI 558532) were cultivated to maturity at the Iowa State University Agronomy Research Farm (Boone, IA). To prevent pollination, developing ear shoots were covered with Lawson #217 shoot bags prior to silk emergence. Whole ears were harvested three days after silks first emerged from encasing husk leaves. Intact ears were transported to the lab on ice. Emerged silks were excised at the site of emergence from the husk leaves. Metabolites were extracted either from silks that were lyophilized and powderized or from fresh silks, as described below.

Lipid extraction experiments were conducted on lyophilized and powderized silks. Approximately twenty immature ears were harvested 3-days post-silk emergence (PSE), and emerged silks were collected and mixed to create a homogenous pool. Pooled silks were stored in a foil pouch, flash frozen in liquid nitrogen, and stored at -80°C. Prior to metabolite extraction, the silks were lyophilized in a freeze-drier system (Labconco) for 48–72 hr at 40 to 50 microbars of vapor pressure. Samples were transferred to conical tubes containing six 5/32” stainless steel beads and were powderized using a Genogrinder2010 (SPEX SamplePrep, NJ).

Lipid extraction experiments were also conducted on fresh and intact silks. Silk samples were pulled from a homogenous pool of emerged silks derived from approximately 20 ears harvested 3-days PSE. Aliquots of approximately 1.3 g of fresh silk were accurately weighed and placed into glass beakers for extraction.

#### Maize seedling experiments

Maize inbred B73 and progeny from a *Gl1/gl1* heterozygote were grown in a ThermoFisher Precision incubator (Model 818) under a diurnal cycle of 16 hr light at 26°C and 8 hr dark at 22°C. Homozygous *gl1* mutant seedlings were identified among the progeny of the *Gl1/gl1* heterozygote by their distinguishing glossy or shiny phenotype, and phenotypically wildtype and *gl1* mutants were separately harvested and analyzed. The second and third leaves were harvested from seedlings 8 days after planting, fresh weights were recorded, and each sample was transferred to a glass beaker and extracted for different time periods, with different solvents. The dry weight of each sample was determined as previously described [22], with the modification that samples were dried at 50°C for 24 hrs.

### Surface lipid extraction and analysis

#### Lipid extraction from lyophilized, powdered silks

**Fig 1:**
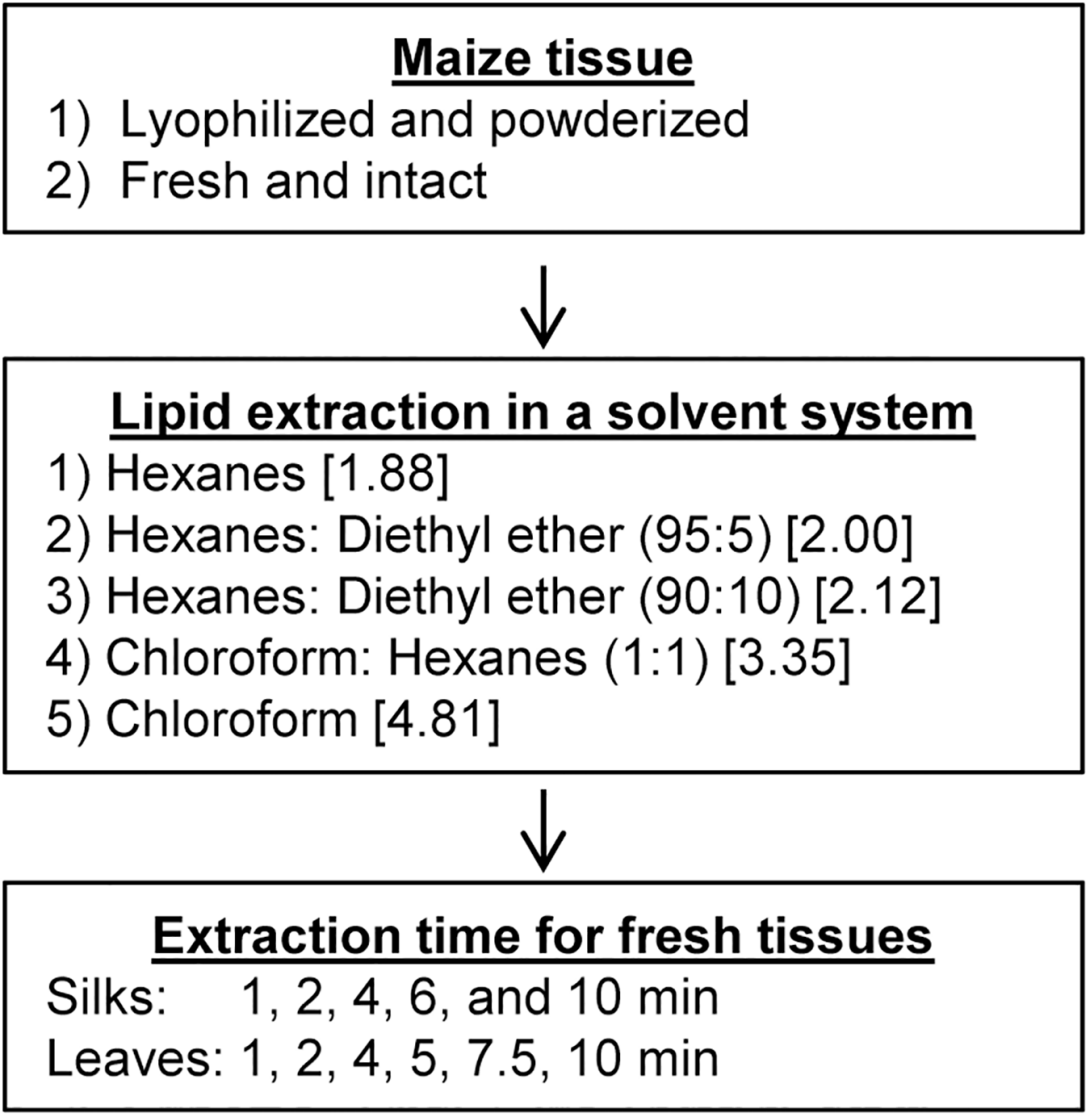
Methods development parameters. Silk tissues were extracted with one of five different solvents for a defined period of time. Solvents are presented in order of increasing polarity, as denoted by the dielectric constants provided in brackets.

Lipid metabolites were extracted using five different solvent systems: 1) pure hexanes; 2) pure chloroform; 3) chloroform:hexanes (1:1, v/v); 4) hexanes:diethyl ether (95:5, v/v); 5) hexanes:diethyl ether (90:10, v/v). Each powdered silk sample (0.03 to 0.11 g) was initially spiked with 10–20 μl of the internal standard (0.5 mg/ml of eicosane) depending on the mass of the sample (10 μl of standard for 0.03 to 0.069 g of sample, or 20 μl of standard for 0.07 to 0.11 g of sample). Solvent (2 ml) from the specified solvent system was added to each sample and the suspension was vortexed for 10 min at 2,500 rpm. Following centrifugation for 5 min at 1,500 x *g*, the extract was transferred to a fresh tube and the pellet was extracted with an additional 3 aliquots of solvent (Fig 1). The pooled extracts for each sample were concentrated under a stream of N_2_ gas in a N-EVAP nitrogen evaporator (Organomation Associates, Inc., MA).

#### Surface lipid extraction from fresh silks and seedlings

Samples of fresh silks (approximately 1.0–1.4 g of fresh tissue) or fresh seedling leaves were extracted either with 15 ml of hexanes:diethyl ether (90:10) or with 15 ml of chloroform, each containing the internal standards, eicosane (1 μg/ml), nonadecanoic acid (1 μg/ml), and heptadecanol (1 μg/ml), for quantification of hydrocarbons, fatty acids and alcohols/aldehydes, respectively. Fresh tissues were immersed in the solvent for a defined period (1 to 10-min). Resultant silk or seedling extracellular lipid extracts were decanted into clean glass tubes and concentrated under a stream of N_2_ gas.

#### Chemical derivatization of extracts

Lipid extracts were derivatized to methyl esters prior to GC-MS analysis as previously described [23], with some modifications. Stated briefly, each lipid extract was derivatized in 2 ml of methanolic HCl (1N) for 1 hr at 80°C. Subsequently, 2 ml of 0.9% (w/v) aqueous NaCl was added and the lipids were then extracted by vortexing at 2,500 rpm for 1 min with 1 ml of hexanes. The organic layer was recovered after centrifugation at 1,000 x *g* for 5 min. To enhance recovery of lipids, the aqueous layer was extracted a second time with hexanes and the recovered extracts were pooled. Samples were concentrated under a stream of N_2_ gas.

After trans-methylation, silylation was performed to derivatize hydroxyl groups that remained underivatized after methylation of the surface lipid extracts (i.e. alcohols, as well as fatty acids that were not transformed to methyl esters during trans-methylation). Dried extracts were dissolved in 0.2 ml of silylation reagent [25:1 acetonitrile:BSTFA (N,O-bis(trimethylsilyl)trifluoroacetamide) with 10% trimethylchlorosilane catalyst (v/v)] and incubated for 30 min at 65°C. Silylated products were concentrated under a stream of N_2_ gas and immediately analyzed via GC-MS.

#### Gas chromatography-mass spectrometry

Prior to GC-MS analysis, the methylated and silylated samples were reconstituted in hexanes. Gas chromatography was performed with an HP-5MS cross-linked (5%) diphenyl (95%) dimethyl polysiloxane column (30 m in length; 0.25-mm inner diameter) using helium as the carrier gas, and an Agilent Technologies series 6890 gas chromatograph, equipped with a model 5973 mass detector (Agilent Technologies, http://www.home.agilent.com). For samples that were trans-methylated only, 1-μl aliquots were injected into the GC via splitless injection, and the GC oven temperature program was as follows: start at 120°C, increase at a rate of 22°C/min to 150°C, increase at a rate of 15.70°C/min to 260°C, increase at a rate of 8.64°C/min to 300°C, increase at a rate of 5°C/min to 320°C and hold at this temperature for 3 min. For samples that were both trans-methylated and silylated, 2-μl aliquots were injected into the GC via splitless injection, and the GC oven temperature program was as follows: start at 100°C, increase at a rate of 10°C/min to 270°C and hold for 2 min, increase at a rate of 3°C/min to 290°C and hold for 2 min, increase at a rate of 5°C/min to 320°C and hold at this temperature for 2 min.

Retention indices of lipid metabolites were calculated by calibration with straight chain hydrocarbons ranging in length from 8 to 40 carbons (C8-C40 alkanes calibration standard; Sigma Aldrich). Quantification analysis was performed using the AMDIS software package [24] with aid from the NIST Mass Spectral library (http://webbook.nist.gov/chemistry/) for compound identification. For the GC method associated with trans-methylated samples, the limits of detection and quantitation were 0.0013 μmol/g dry tissue and 0.0042 μmol/g dry tissue, respectively. For the GC method associated with samples that were both trans-methylated and silylated, the limits of detection and quantitation were 0.0031 μmol/g dry tissue and 0.010 μmol/g dry tissue, respectively.

#### Quantitative and statistical methods

All metabolite abundances were quantified according to the initial amount of internal standard used and were reported as μmol of metabolite (or metabolite class) per g of dry tissue. For experiments conducted on dry silks as well as fresh silks and seedlings, quantification was performed relative to the dry weight of that tissue to eliminate perceived differences in metabolite concentrations due to differences in water content within tissues from different genotypes or conditions. To estimate the dry weights for samples from which extracellular surface lipids were extracted, a conversion factor was calculated from the dry and fresh weights from ten additional silk samples, using the formula: DW/FW ratio = average dry weight/average fresh weight. All fresh weights of samples that were used for lipid extraction were multiplied by the DW/FW conversion factor to estimate the dry weight.

Unless otherwise indicated, data were generated from a minimum of three biological replicates and average abundances and standard errors are reported. Abundances for individual metabolites identified in each experiment are reported in Supporting Information, S1–S6 Tables. Data were analyzed via ANOVA and Tukey’s Honest Significant Difference (HSD) test (JMP Pro 11, SAS Institute Inc., Cary, NC). In all cases, statistical comparisons are made only among samples collected within a single experiment.

## Results and discussion

This study establishes a method for extracting extracellular surface lipids from plant tissues (i.e. maize silks and seedling leaves), with specific focus on 1) the choice of solvent for efficient extraction of non-polar and amphipathic metabolites of different polarities, and 2) extraction time. In a previous characterization of extracellular surface lipids from maize silks, it was demonstrated that hydrocarbons primarily accumulate on the silk surface and internal accumulation was not detectable [20]. Based on this observation, hydrocarbon content reported by Perera and colleagues [20] was measured from silks that were first lyophilized and ground into a fine powder, and then extracted with hexanes and subsequently purified by treating with silica that would absorb more polar compounds (e.g. sugars and fatty acids). In the current study, we have developed methodology to extract and characterize non-polar and amphipathic metabolite classes that are frequent constituents of extracellular surface lipids in plants (e.g. hydrocarbons, fatty acids, aldehydes, alcohols) using a single and straightforward extraction procedure that is amenable to large sample sizes and can be applied to fresh, intact tissues. All method-development experiments were conducted on tissues from the maize inbred, B73. The solvent- and extraction time-optimized method was applied to the analysis of silks from maize inbreds B73 and Mo17, and on *glossy1* seedlings that are impaired in extracellular surface lipid accumulation.

### Comparison of solvent systems for extraction of extracellular surface lipids from fresh silks

**Fig 2:**
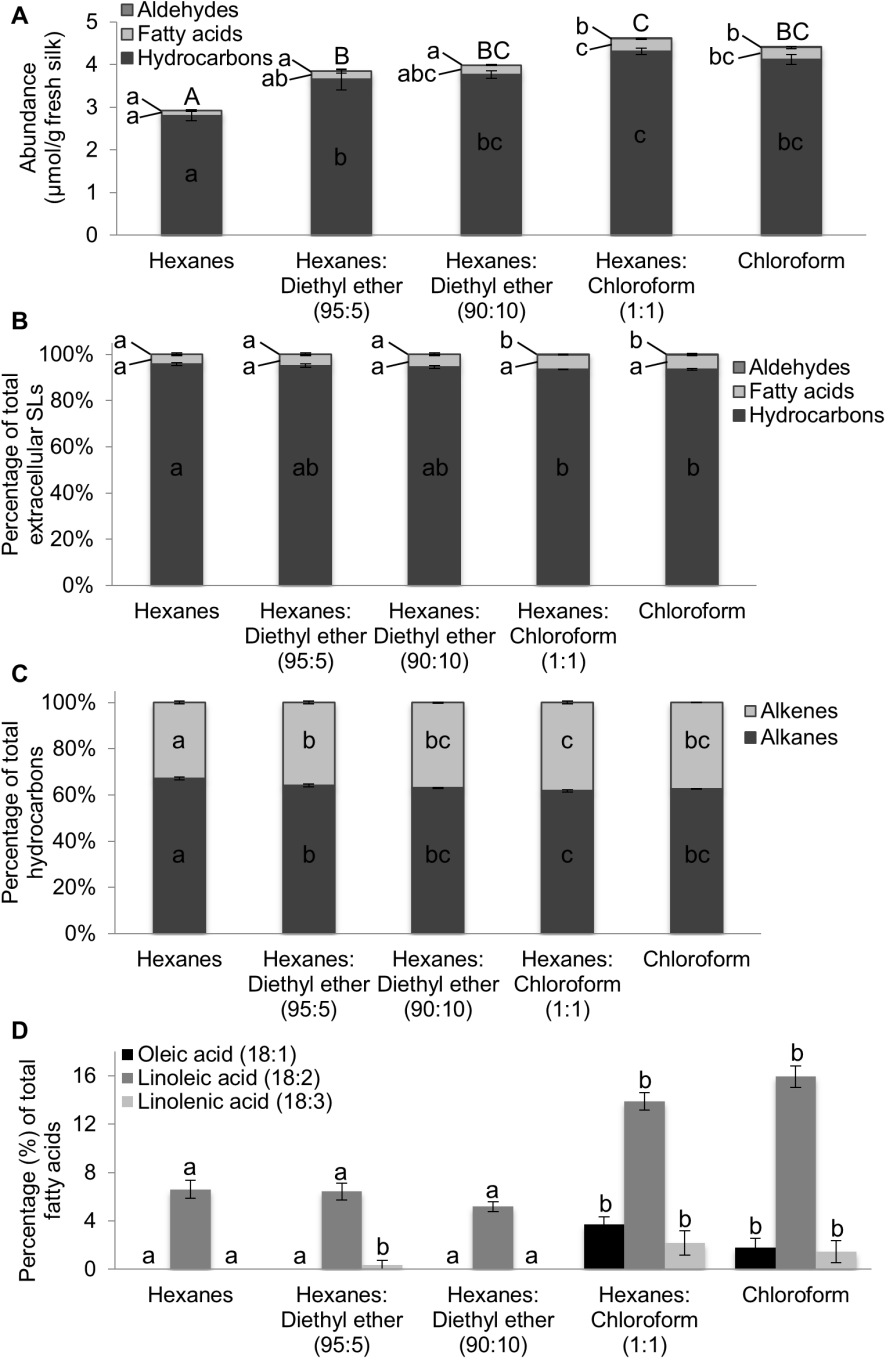
Comparison of lipid recovery from fresh, intact silks with different extraction solvents. A. Total extracellular surface lipid recovery (including hydrocarbons, fatty acids and aldehydes) from fresh, emerged silks of inbred B73 extracted for four minutes with the specified solvents: hexanes, hexanes:diethyl ether (95:5 and 90:10), chloroform:hexanes (1:1) and chloroform. B. Relative abundances (%) of observed extracellular surface lipid classes. C. Relative abundances (%) of saturated (alkanes) and unsaturated (alkenes) relative to total hydrocarbon recovery. D. Relative abundances (%) of recovered unsaturated 18-carbon fatty acids relative to total fatty acid recovery. Different lowercase letters above or within data-bars for a specific lipid class or constituent denote statistically significant differences in recovery with different solvents, at p<0.05, according to a Tukey’s HSD test. In panel A, uppercase letters denote statistically significant differences among total extracellular surface lipid recoveries. A minimum of five replicates were used per solvent, with an N = 29. Averages ± SE are reported.

Five solvent systems were selected based on their range of polarities, as defined by their dielectric constants (Fig 1). Extraction solvents included chloroform and hexanes, solvent systems previously and routinely used to extract extracellular surface lipids [19–21], and three additional solvent systems [chloroform:hexanes (1:1); hexanes:diethyl ether (95:5); and hexanes:diethyl ether (90:10)]. Total surface lipid recovery varied approximately 1.6-fold between the solvent with the highest recovery, chloroform:hexanes (1:1), and the solvent with the least recovery, hexanes (Fig 2A; concentrations of each individual constituent are reported in S1 Table). Extraction of fresh, intact silks yielded a high abundance of hydrocarbons (93–96% of total surface lipids), confirming that hydrocarbons are the predominant class of surface lipid constituents.

Recovery of the hydrocarbon and fatty acid classes of extracellular surface lipids from fresh, intact silks varied among the different solvent systems evaluated. Extraction of fresh, intact silks with the hexanes solvent resulted in variation in total hydrocarbon recovery from 2.805 μmol/g dry tissue when extracted in hexanes to 4.3244 μmol/g dry tissue when extracted in the hexanes: chloroform (1:1) solvent system (S2 Table). However, the relative abundances of hydrocarbons did not statistically differ among the hexanes and hexanes:diethyl ether solvents (Fig 2B). The relative recovery of unsaturated hydrocarbons (alkenes) was reduced in the hexanes solvent, albeit this was minor (i.e. 6%; p-value<0.0001), as compared to the other solvent systems (Fig 2C). Extraction with chloroform, chloroform:hexanes (1:1), and both hexanes:diethyl ether solvents yielded similar relative recovery of alkenes, ranging between 36 and 38% of total extracted extracellular surface lipids.

In contrast, the relative abundance of unsaturated fatty acids, among all fatty acids extracted, varied more significantly (~25%) among solvents (ANOVA R^2^ = 0.79, p-value<0.0001). Greatest efficiency was observed in the chloroform-containing solvents (approx. 20% of total fatty acids) and lowest efficiency in the hexanes and hexanes:diethyl ether (90:10) solvents (approx. 7%). This variation in the relative recovery of unsaturated fatty acids was due to increased recovery of oleic (C18:1), linoleic (C18:2) and linolenic (C18:3) acids in the chloroform-containing solvents (Fig 2D). For example, the relative abundance of linoleic (C18:2) acid comprised 16% of fatty acids in chloroform extracts, whereas only approx. 5% in the hexanes: diethyl ether (90:10) solvent. Similarly, oleic (18:1) and linolenic (18:3) acids were recovered in chloroform-containing extracts, but were either not detected or were observed at low levels in the hexanes and hexanes:diethyl ether solvent systems. These specific unsaturated fatty acid constituents, in addition to palmitic acid (C16:0), were the most abundant chloroform-soluble fatty acids extracted from powdered silks in a previous study [20], suggesting that these fatty acid species might be derived from internal lipid pools and they are more readily extractable using the more polar, chloroform-containing solvents.

**Fig 3:**
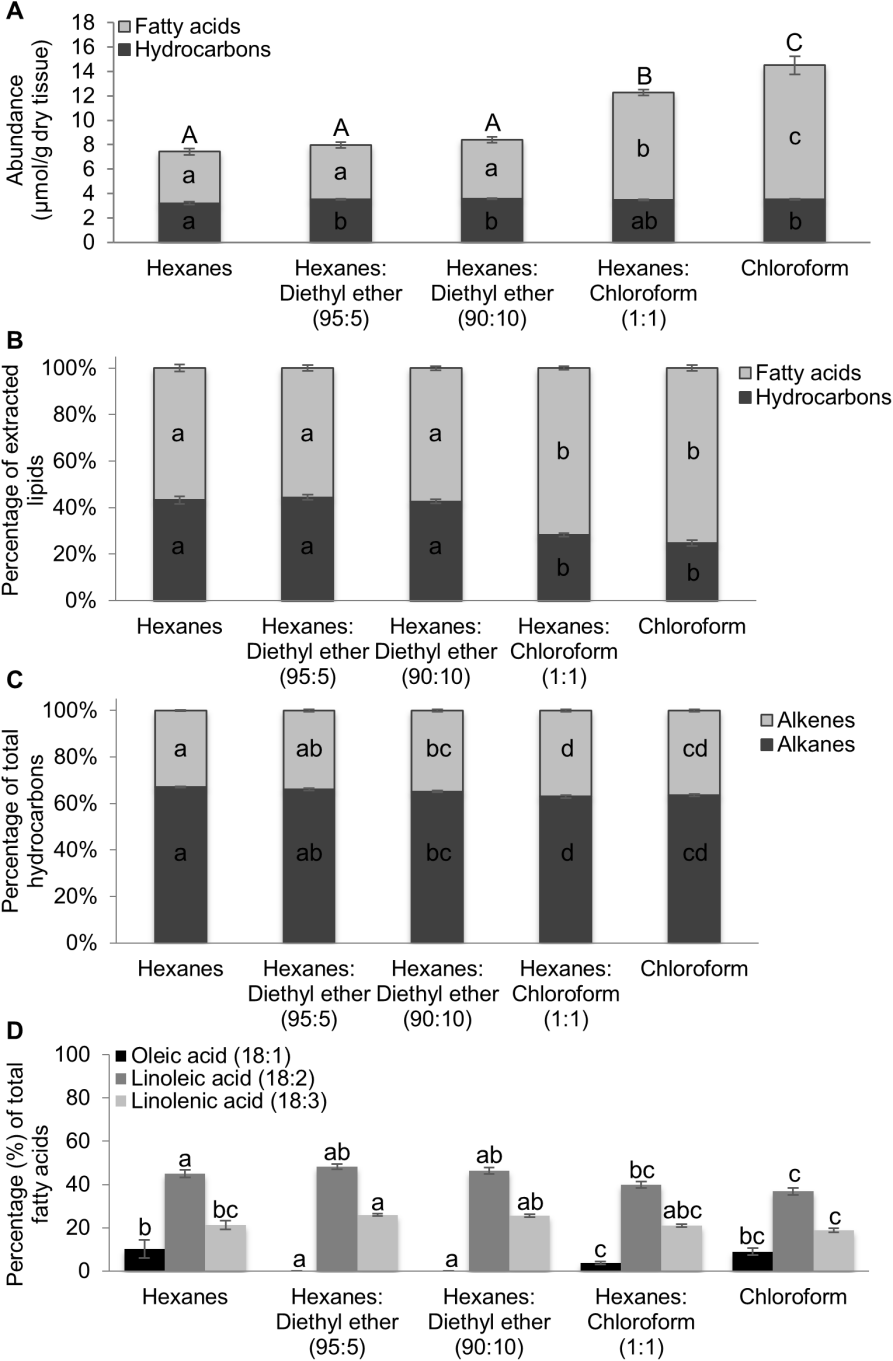
Comparison of lipid recovery from lyophilized, powderized silks extracted with different solvents. A. Total extracted lipid recovery (including hydrocarbons, fatty acids, aldehydes) from lyophilized, powderized emerged silks of inbred B73 extracted for ten minutes with the specified solvents: hexanes, hexanes:diethyl ether (95:5 and 90:10), chloroform:hexanes (1:1) and chloroform. B. Relative abundances of the observed lipid classes, hydrocarbons and fatty acids. Aldehydes were not detected above the limit of quantitation in this experiment. C. Relative abundances (%) of saturated (alkanes) and unsaturated (alkenes) relative to total hydrocarbon recovery. D. Relative abundances (%) of unsaturated 18-carbon fatty acids relative to total fatty acid recovery. Different lowercase letters above or within data-bars for a specific lipid class or constituent denote statistically significant differences in recovery with different solvents, at p<0.05, according to a Tukey’s HSD test. In panel A, uppercase letters denote statistically significant differences among total extracellular surface lipid recoveries. A minimum of four replicates were used per solvent, with an N = 28. Averages ± SE are reported.

To test the hypothesis that the chloroform-containing solvents extract fatty acids from internal lipid pools, the efficiency of lipid extraction across the five solvent systems was tested on inbred B73 emerged silk samples that were lyophilized and powderized, which provided more direct access to internal lipid pools. The extracted hydrocarbons and fatty acids were assessed via GC-MS after derivatization of carboxyl groups to methyl esters (Fig 3; concentrations of individual constituents are reported in S2 Table). As with fresh silks, lipid recovery was highest with the chloroform and chloroform:hexanes (1:1) solvents. Total recovery with chloroform was approximately double of that with hexanes (Fig 3A), with 90% of the observed variation in total lipid concentration attributable to solvent choice (ANOVA R^2^ = 0.90, p<0.0001). The increased recovery obtained with the chloroform-containing solvent systems is attributable to the enhanced extraction of fatty acids (Fig 3A), which comprise approximately 75% of total lipids in the chloroform-containing solvents as compared to 55% fatty acids obtained with the hexanes:diethyl ether or hexanes-only solvents (Fig 3B). In contrast to fatty acid recovery from lyophilized B73 emerged silks, with the exception of the hexanes solvent, total hydrocarbon recovery did not differ significantly among the different solvent systems tested (Fig 3A). Using hexanes as a solvent yielded approximately 10% lower recovery of hydrocarbons as compared to each of the other solvents (ANOVA R^2^ = 0.47, p-value = 0.005; Fig 3A). Examination of the saturated and unsaturated metabolites within the hydrocarbon class (i.e. alkanes and alkenes, respectively) reveals minor, yet statistically significant, variations in recovery across the five solvents (ANOVA R^2^ = 0.72, p-value<0.0001). The relative abundance of unsaturated hydrocarbons (alkenes) relative to total hydrocarbons was lowest with the non-polar hexanes-only solvent and highest with the more polar, chloroform-containing solvents; however, variation among solvents was only 4% (Fig 3C). Results presented herein suggest that solvents of higher polarity, containing either diethyl ether or chloroform, yield slightly higher recovery of hydrocarbons than the hexanes solvent system previously used to evaluate hydrocarbon accumulation on maize silks [20], apparently due to increased extraction of unsaturated hydrocarbons.

The higher recovery of fatty acids observed from powdered silks is expected, since internal lipids as well as extracellular surface lipids are accessible to extraction when pulverized tissue is used. However, the increased recovery of the fatty acids in chloroform-containing solvents as compared to the hexanes:diethyl ether or hexanes-only solvents is likely due to the increased polarity of the former compared to the latter. Across solvent systems tested, the predominant unsaturated fatty acids identified from silk extracellular surface lipids (oleic, linoleic and linolenic acids; Fig 2D) collectively comprise approximately 65–75% of total fatty acids extracted from lyophilized, powdered silks (Fig 3D). Interestingly, the relative abundances of linoleic and linolenic acids extracted with the hexanes and hexanes:diethyl ether solvent systems are similar to or slightly higher than when extracted with the chloroform containing solvents. This is due to increased recovery of saturated fatty acids, particularly palmitic (C16:0) and stearic (C18:0) acids, observed with the chloroform containing solvents (S2 Table), which results in a higher relative abundance of saturated fatty acids. The amounts of fatty acid constituents present in the powdered samples extracted with either chloroform or chloroform:hexanes are similar to fatty acid levels observed in homogenized silks extracted via the Bligh & Dyer method, which extracts both free fatty acids and fatty acids esterified to different lipids [20].

Collectively, the extraction experiments conducted on fresh, intact silks, as opposed to lyophilized silks, demonstrate that each of the tested solvents extract total hydrocarbons to a similar extent, with the exception of hexanes (Fig 2A, Fig 3A). However, fatty acid recovery, and unsaturated fatty acid recovery in particular, varies considerably as compared to lyophilized, powdered silks. The data suggest that perhaps chloroform has an enhanced ability to extract leaf-internal fatty acid constituents and therefore use of this solvent could complicate the interpretation of metabolite profiling data obtained from plant surfaces. In contrast, the hexanes:diethyl ether solvent systems were identified as solvents that efficiently extracted hydrocarbons with reduced recovery of potentially internal metabolites.

### Optimization of extraction time for extracellular surface lipids of fresh silks

In the solvent tests described above, biological samples were each extracted for a standard 4-minute extraction period. We next tested the effect of different times of extraction, with the most promising solvent identified and compared to the commonly used solvents, pure chloroform and pure hexanes. The hexanes:diethyl ether (90:10) solvent system was chosen for this testing, based on its slightly higher polarity (compare dielectric constants, Fig 1) and therefore its potential ability to extract amphipathic compounds (e.g. aldehydes, fatty acids). To optimize the time of extraction for fresh silks from maize inbred B73, duration of extraction was tested for 1, 2, 4, 6, and 10 min for each solvent, and silk samples were randomly assigned a solvent type and extraction time. The concentrations of each extracted constituent are reported in S3 Table.

**Fig 4:**
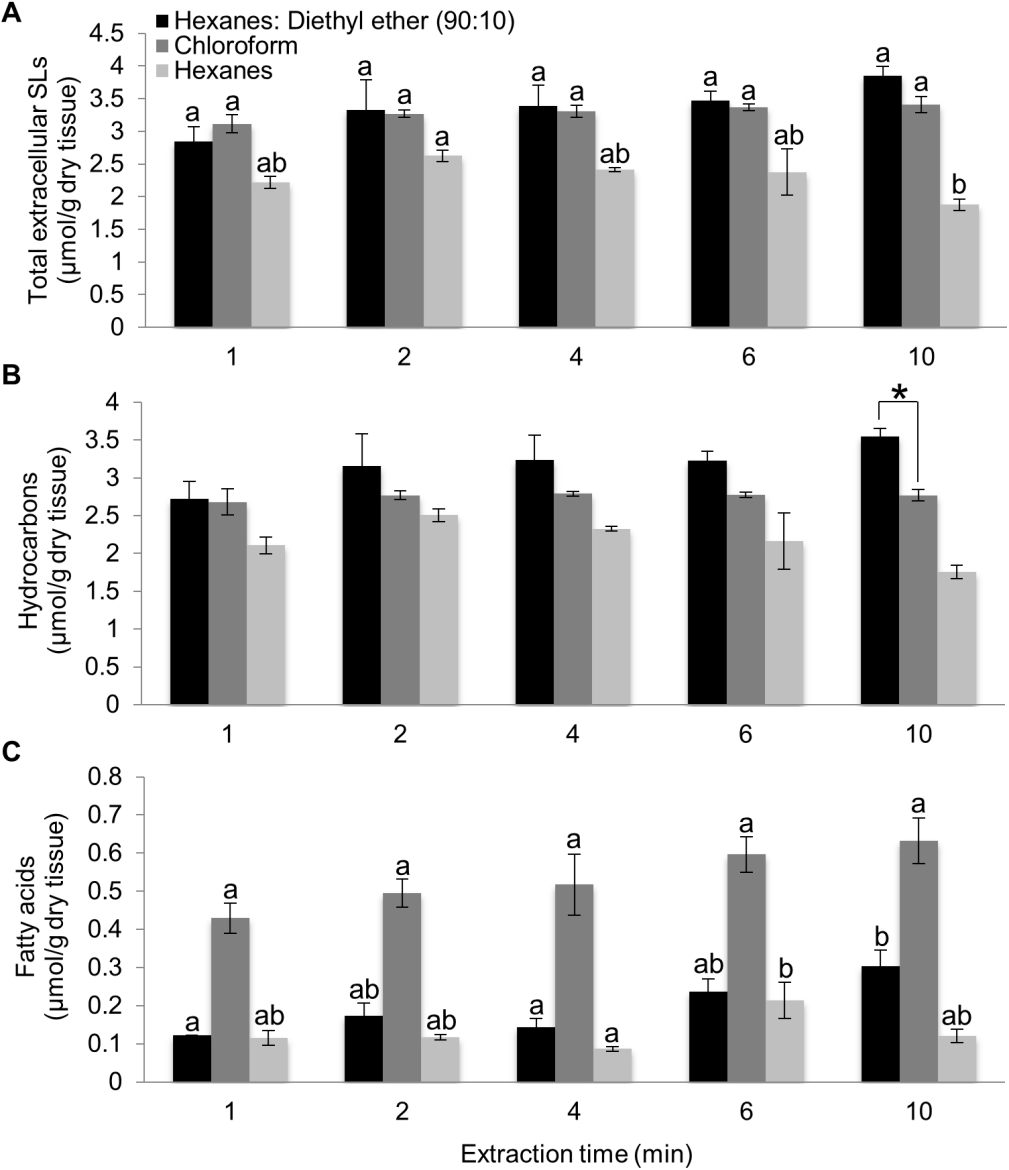
Optimization of extraction time for the recovery of extracellular surface lipids from silks. Recovery of total extracellular surface lipids (A), hydrocarbons (B), and fatty acids (C) from emerged maize silks of inbred B73 extracted for different periods of time with hexanes:diethyl ether (90:10), chloroform or hexanes. Aldehydes comprised <1% of observed metabolites, regardless of extraction period and therefore are not shown. The asterisks denote statistically significant differences in recoveries with different solvents, at p<0.05, according to a Tukey’s HSD test. In panels A and C, different letters above data-bars denote statistically significant differences in recoveries in response to time of extraction with a specific solvent, at p<0.05. No such differences were observed in the data presented in panel B. A minimum of three replicates were used per combination of extraction time and solvent type, with an N = 67. Averages ± SE are reported.

The recovery of total extracellular surface lipids (Fig 4A) and hydrocarbons (Fig 4B) did not differ significantly among extraction times for either the chloroform or the hexanes:diethyl ether (90:10) solvents, with the exception of hydrocarbon recovery from a 10-min extraction (Fig 4B). In comparison to the solvents with higher polarities (i.e. chloroform and hexanes:diethyl ether (90:10) solvents), the hexanes solvent exhibited the lowest recovery at each extraction time and was statistically different than either the chloroform or hexanes:diethyl ether (90:10) solvent at each time point (p<0.03). These results are consistent with observations from the initial solvent test experiments that were conducted with an extraction time of 4 min (Fig 2A). Based on these results, the remainder of the manuscript will focus on the comparison of the hexanes:diethyl ether (90:10) and chloroform solvent systems.

Fatty acid recovery is impacted both by the solvent type and by extraction time (ANOVA R^2^ = 0.83, p-value<0.0001). As compared to the hexanes:diethyl ether (90:10) solvent, fatty acid recovery with chloroform extraction yields ~3.5-fold higher concentrations at short extraction times (1, 2 and 4 min) and ~3-fold higher concentrations after a 10-min extraction (Fig 4C). At each of these extraction times, the primary fatty acid constituents present in the chloroform extracts were palmitic acid, stearic acid, linoleic acid and linolenic acid. As discussed earlier, these are key fatty acid constituents of leaf-internal lipids, as observed in extracts from lyophilized silks (Fig 3), and also demonstrated previously from emerged silks from the inbred B73 [12]. Indeed, it has been noted that free palmitic and stearic acid metabolites present in internal lipid pools are potential extraction artifacts [4, 25]. Moreover, exposure of silks to chloroform, even with an extraction time of just 1 minute, resulted in marked discoloration of the silks (S1 Fig), whereas little discoloration was observed for silks extracted in hexanes:diethyl ether (90:10), even at an extraction time of 4 min (S1 Fig). Collectively, these data suggest that even at very short time intervals, it is possible that the chloroform solvent is extracting both the intended extracellular surface lipids, as well as unintended internal metabolites.

Within-solvent comparisons illustrate that major surface lipid constituent classes are recovered in similar concentrations across extraction times from 1 to 6 min (Fig 4B and 4C). Only a few minor constituents showed a statistically significant, albeit minor, increase in extractability at 2 or 4 minutes, as compared to 1 min. Therefore, a 2 or 4-minute extraction in hexanes:diethyl ether (90:10) is suggested for the stable extraction of surface hydrocarbon and fatty acid constituents, with reduced potential for concomitant extraction of potential internal lipids. From a technical standpoint, an additional advantage of the 4-min extraction, compared to shorter extraction times, is that it allows for staggered extraction of multiple samples at one time, and hence more rapid extraction of large sample numbers, making the procedure more amenable to large-scale studies (e.g. quantitative genetic approaches).

### Application of optimized method to extraction of leaf extracellular surface lipids

**Fig 5:**
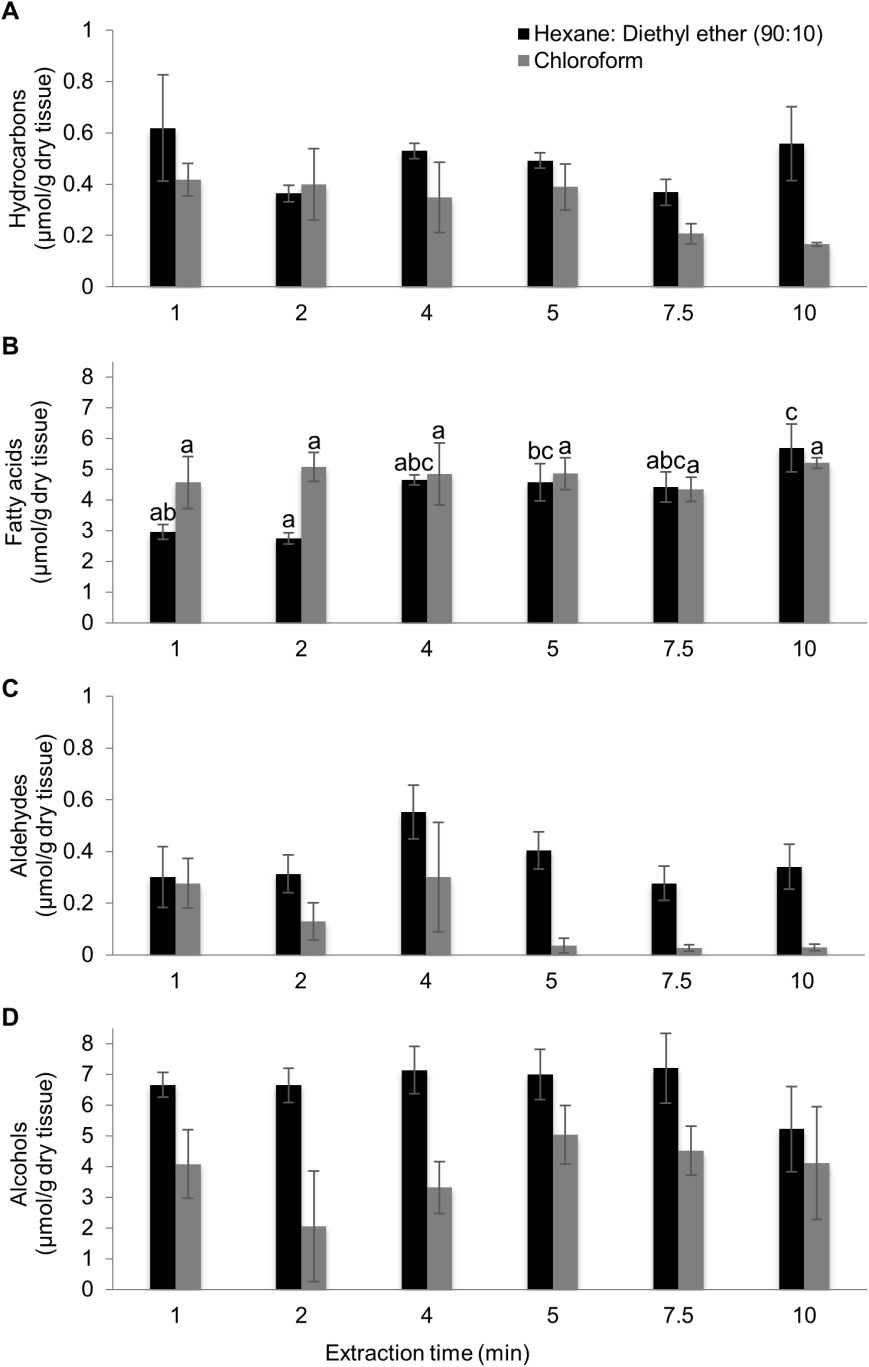
Optimization of extraction times for recovery of extracellular surface lipids from seedlings. Recovery of hydrocarbons (A), fatty acids (B), aldehydes (C), and alcohols (D) from seedling leaves of inbred B73 treated with hexanes:diethyl ether (90:10) or chloroform for different extraction times. Different letters above data-bars in panel B denote statistically significant differences in recoveries in response to time of extraction, at p<0.05, according to a Tukey’s HSD test. No such differences were observed for the data presented in panels A, C and D. Four replicates were used per combination of extraction period and solvent type, with an N = 48. Averages ± SE are reported.

The effectiveness of extraction of non-polar and amphipathic extracellular surface lipids from maize seedling leaves was tested using the traditional chloroform solvent system and the optimized hexanes:diethyl ether (90:10) solvent system suggested by this study. Seedling leaves were randomly assigned a solvent type and an extraction time (i.e. 1, 2, 4, 5, 7.5 and 10 min). The concentrations of each extracted constituent are reported in S3 Table. Extracellular surface lipids from inbred B73 seedlings are comprised primarily of alcohols ranging in lengths from 16 to 32 carbons, fatty acids ranging in lengths from 16 to 26 carbons (Fig 5, S4 Table), and, to a lesser extent, a single aldehyde of 32-carbons (C32) in length (i.e. dotriacontanal). These data are consistent with previously published surface lipid profiles from seedling leaves of several different inbred lines [22, 26–29].

Extraction time impacted surface lipid recovery for seedling leaves extracted in the hexanes:diethyl ether (90:10) solvent system (Fig 5). Although recoveries of hydrocarbons (Fig 5A), aldehydes (Fig 5C) and alcohols (Fig 5D) were not impacted by extraction time in either solvent, fatty acid recovery was significantly impacted by extraction time in hexanes:diethyl ether (90:10) (R^2^ = 0.87, p-value<0.001; Fig 5B). Total fatty acids approximately doubled when extracted for 4–7.5 min in hexanes:diethyl ether (90:10), as compared to 1 or 2 minute extractions (p-value<0.005). Extraction for 10 min, however, resulted in an approx. 60% increase in fatty acid recovery as compared to shorter extraction times. This is potentially due to extraction of leaf-internal fatty acids, with substantial increase in the recovery of palmitic acid, linoleic acid and linolenic acid.

In comparison, extraction time had a smaller impact on lipid recovery when using chloroform as the extraction solvent, with total surface lipid recovery not impacted by extraction time (R^2^ = 0.08, p-value = 0.93).

**Fig 6:**
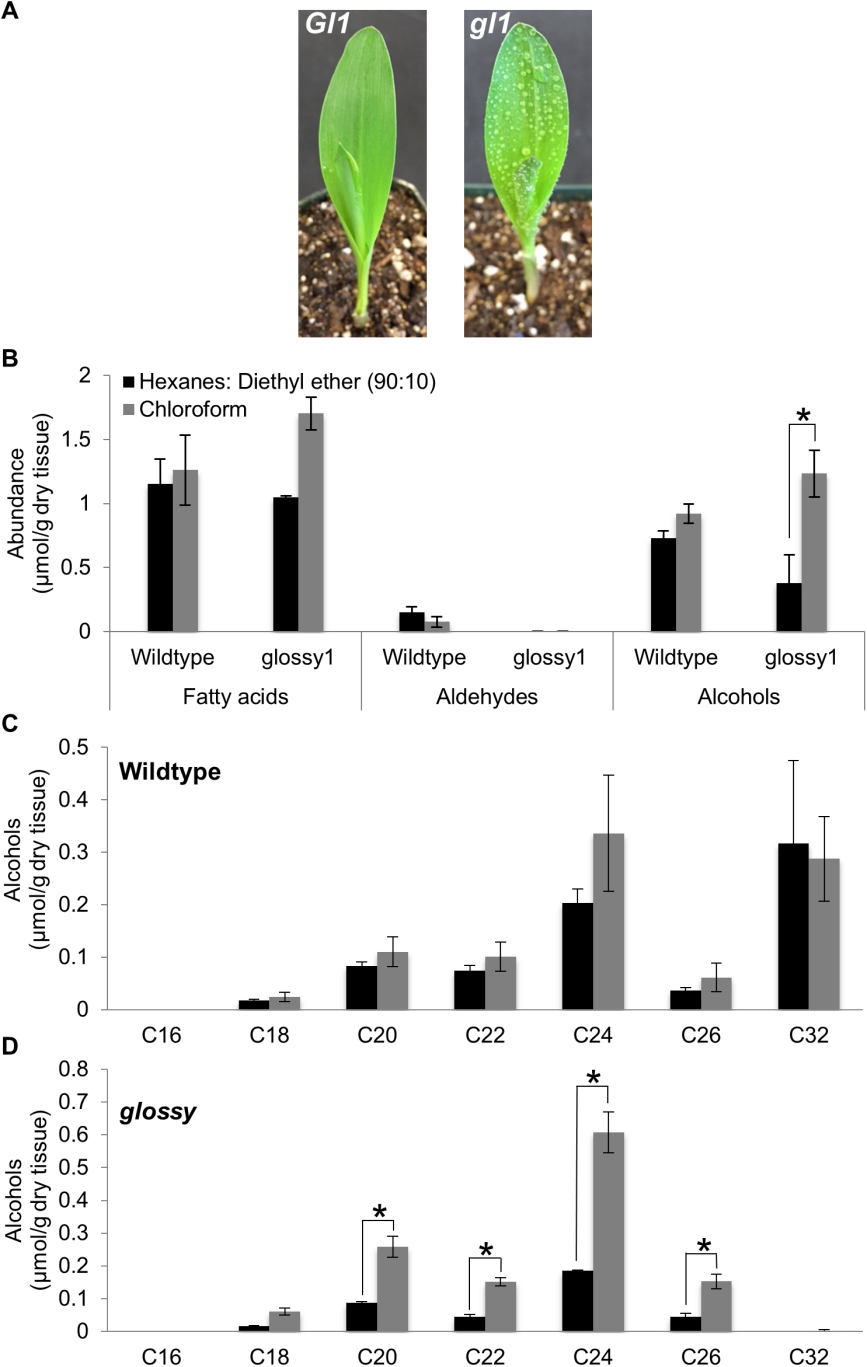
Evaluation of extracellular surface lipids from wildtype (*Gl*) and *glossy1* (*gl1*) seedlings. A. The water adherence phenotype in *gl1* seedlings impaired in surface lipid deposition. B. Extracellular surface lipids (fatty acids, aldehydes and alcohols) extracted from wildtype and *gl1* seedlings, using chloroform or hexanes:diethyl ether (90:10) solvent systems. C. Individual alcohol constituents of different carbon-chain lengths, extracted from wildtype seedlings with hexanes:diethyl ether (90:10) or chloroform. D. Individual alcohol constituents of different carbon-chain lengths, extracted from *gl1* seedlings with hexanes:diethyl ether (90:10) or chloroform. Asterisks denote statistically significant differences between different solvents in the recovery of specific constituents, at p<0.05, according to a Tukey’s HSD test. A minimum of two replicates were used per combination of genotype and solvent type, with an N = 13. Averages ± SE are reported.

To demonstrate the utility of the hexanes:diethyl ether (90:10) solvent system for distinguishing surface lipid profiles of wildtype seedlings vs. seedlings deficient in extracellular surface lipids, this solvent system and the chloroform solvent were both used for extracellular surface lipid extraction of *glossy1* (*gl1*) mutants and wildtype siblings. Homozygous *glossy1* mutants are defective in extracellular surface lipid accumulation on seedling leaves [30] and are recognized by their shiny, or glossy, phenotype [31], as well as the propensity for water droplets to adhere to the seedling leaf surface (Fig 6A). Based on the assessment of extraction time on metabolite profiles (Fig 5), a 2-min extraction was applied with both solvents. Because esters were shown to comprise only 1% of extracellular surface lipids from seedling leaves of inbred B73 [22], individual extracts were derivatized via trans-methylation followed by silylation, which converts this minor fraction of esters to the component fatty acid (fatty acyl methyl ester) and alcohol (silyl ether) constituents. Concentrations of each individual constituent for this experiment are reported in S5 Table, and representative chromatograms for wildtype and *gl1* seedling leaves extracted with either of the two solvents are depicted in S2 Fig.

By comparing extraction solvents of different polarities, we can assess differential impact on lipid recovery from seedlings with different extracellular surface lipid loads (Fig 6B). For both solvent systems, recovery of dotriacontan-1-ol (i.e. C32 alcohol) from the *gl1* mutant was reduced by 50- to 80-fold as compared to wildtype (Fig 6C and Fig 6D). Moreover, dotriacontanal, the C32 aldehyde, which has been shown to comprise approximately 96% of the extracellular aldehydes [26], was undetectable in *gl1* seedlings regardless of extraction solvent. These findings are consistent with previous characterization of the *gl1* mutant [27, 30], in that dotriacontan-1-ol and dotriacontanal accumulation were greatly reduced in *gl1* mutants as compared to wildtype seedlings.

Although total alcohol, total fatty acid and total aldehyde concentrations did not significantly differ between wildtype extracts obtained with either chloroform or hexanes:diethyl ether (90:10) extraction, significant differences were observed between these solvents for *glossy1* mutants. For example, fatty acid recovery was enhanced by approximately 60% in *gl1* seedlings extracted in chloroform as compared to extraction in hexanes:diethyl ether (90:10) (p-value<0.05). Similar to fatty acid recovery in the *gl1* mutant, recovery of alcohols was increased (approx. 200%, Fig 6B) due to increases in specific alcohol constituents (i.e. C20, C22, C24 and C26 alcohols) in the *gl1* mutant when extracted with chloroform (compare Fig 6D and Fig 6C). Together, the enhanced recoveries of certain fatty acid and alcohol constituents in *gl1* seedlings extracted with chloroform could potentially be attributed to the ability of the chloroform solvent to more readily penetrate the leaf surface and extract metabolites of varying chain lengths from internal tissues in the *gl1* mutant, which is deficient in key extracellular surface lipids (i.e. C32 alcohol and aldehyde).

The above findings suggest the importance of extraction solvent considerations when evaluating the chemotypic effects of mutations on fatty acid biosynthesis and extracellular surface lipid composition.

### Application of optimized method to emerged and husk-encased silks from two maize genotypes

**Fig 7:**
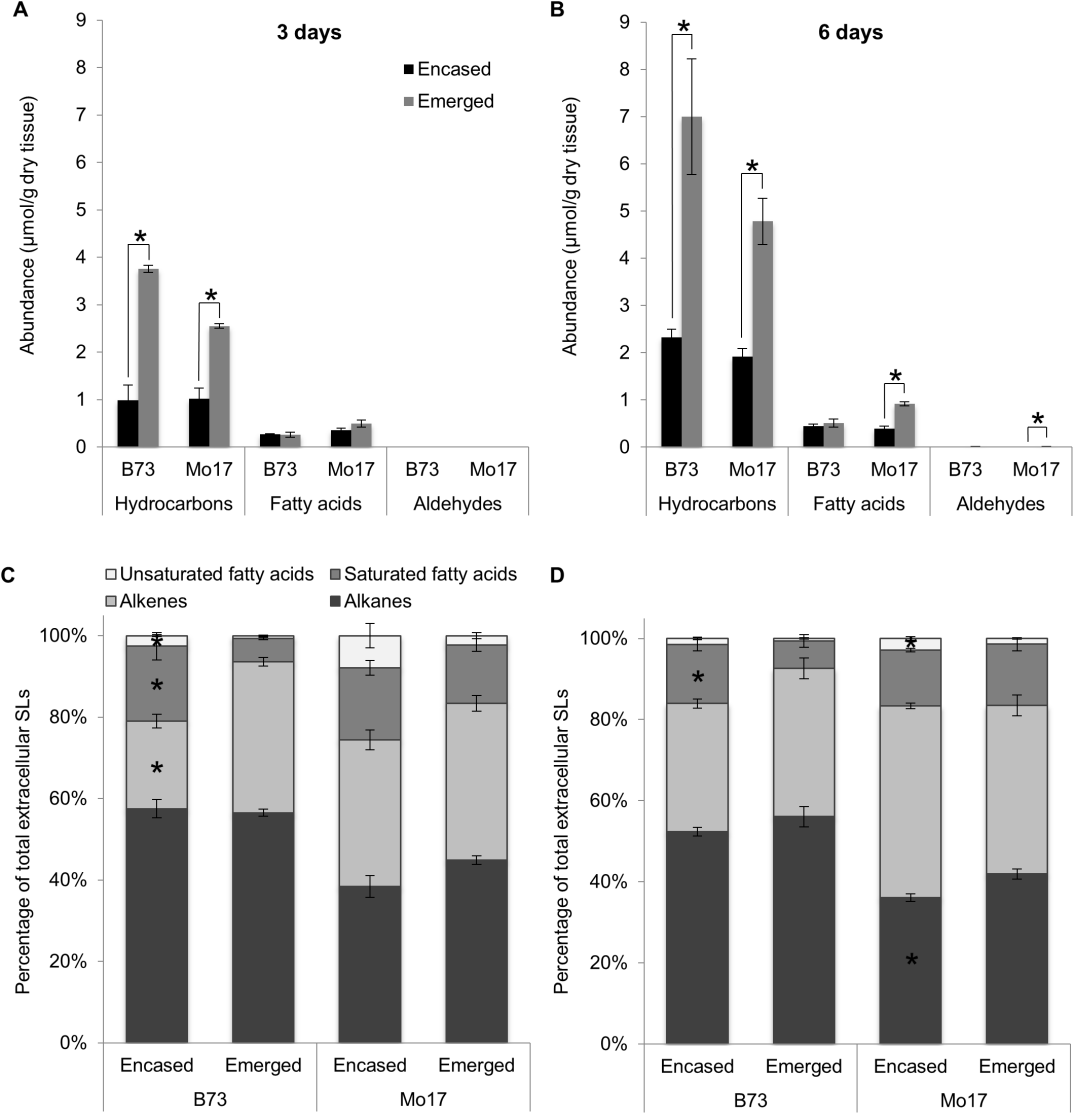
Evaluation of extracellular surface lipids from B73 and Mo17 silks harvested 3- and 6-days post-silk emergence. A and B. Concentrations of extracellular surface lipid classes (hydrocarbons, fatty acids and aldehydes) from silks harvested at either 3 days (A) or 6 days (B) post-silk emergence (PSE). Surface lipid concentrations are compared between silks that were encased by the husk leaves (black square) and silks that had emerged from the husk leaves (grey square). C and D. Relative abundances (%) of alkanes (saturated), alkenes (unsaturated), and saturated and unsaturated fatty acids relative to total extracellular surface lipids on silks harvested either 3 days (C) or 6 days (D) PSE. Relative abundances are shown as stacked bars and are compared between emerged and husk-encased silks. In all cases, aldehydes comprised <1% of observed metabolites and therefore are not shown. For panels A-D, an asterisk denotes statistically significant differences between these two samples, at p<0.05 according to a Tukey’s HSD test. Four replicates were used per combination of genotype, emerged vs. husk-encased silks and days PSE, with an N = 32. Averages ± SE are reported.

To dissect the dynamic changes in the surface lipid metabolome that occur in response to silk age or silk environment, the optimized extraction method was applied to silks harvested either three- or six-days post-silk emergence (PSE) in two agronomically important maize inbreds (B73 and Mo17). Furthermore, these analyses compared silks that had either emerged from the husk leaves into the external environment (i.e. the emerged fraction) to silks that were still encased by the husk leaves (i.e. the husk-encased fraction). With both genotypes, and irrespective of whether the silks were emerged or husk-encased, the extracted extracellular surface lipids were comprised predominantly of hydrocarbons and fatty acids (Fig 7A and 7B), with quantifiable, albeit trace, amounts of aldehydes, which were primarily detectable in emerged silks at 6-days PSE (Fig 7B, S6 Table). Quantitatively, total extracellular surface lipids recovered from emerged silks collected 3-days PSE were 3.2-fold higher for B73, and 2.2-fold higher for Mo17 as compared to husk-encased silks from each inbred. This was largely due to a 3.8-fold and 2.5-fold increase in hydrocarbon accumulation in emerged versus husk-encased silks of inbreds B73 and Mo17, respectively (Fig 7A). These findings are consistent with a previous report of a 3- to 5-fold increase in hydrocarbon accumulation on emerged, as compared to husk-encased silks from inbred B73 [20]. In a similar survey of seven maize inbreds, hydrocarbon accumulation was up to 4-fold higher in emerged vs. husk-encased silks harvested from ears at 4-days PSE [21].

With emerged silks harvested 6-days PSE, from either inbred, total surface lipid accumulation was ca. 85% higher as compared to 3-days PSE, which is similar to hydrocarbon accumulation patterns presented for inbred B73 in a previous report [20], and within the range observed for 6-days vs. 3-days PSE shown for select inbreds, ranging from no significant difference to more than a doubling in surface lipid accumulation [21]. Similar to at 3-days PSE, hydrocarbon accumulation on emerged silks for both inbreds at 6-days PSE was approximately 2.5-fold higher than for husk-encased silks (Fig 7B). In summary, relative trends of hydrocarbon accumulation on maize silks were similar using our extraction protocol as compared to previously reported silk surface lipid profiling data [20, 21], demonstrating the efficacy of our selected extraction solvent.

Unlike previous studies [20, 21] that utilized a hexanes solvent, the optimized extraction protocol was able to profile both hydrocarbon and fatty acid accumulation on silks, and differences in fatty acid accumulation were observed between inbred genotypes. With inbred B73, both at 3- and 6-days PSE, fatty acid concentration does not differ between emerged and husk-encased silks (Fig 7A and 7B). However, with inbred Mo17 there is a 2.3-fold increase in fatty acid accumulation on emerged silks as compared to husk-encased silks at 6-days PSE.

Comparison of the relative abundances of fatty acid (saturated and unsaturated) and hydrocarbon (alkanes and alkenes) classes in extracted extracellular surface lipids reveals distinct differences between emerged and husk-encased silks. For example, at 3-days PSE in inbred B73 (Fig 7C), the relative abundance of fatty acids decreases from ~20% of total extracellular surface lipids for husk-encased silks to 6% for emerged silks (p<0.01). Similarly, at 6-days PSE fatty acids comprise 16% of total extracellular surface lipids in emerged silks and only 8% in husk-encased silks (p-value<0.01; Fig 7D). In contrast, on Mo17 silks the relative abundance of total fatty acids does not statistically differ between emerged and husk-encased silks, either at 3- or 6-days PSE and remains between 17 and 26% of total extracellular surface lipids. The relative abundances of fatty acids in both B73 and Mo17 is within a similar range as reported by a previous study of extracellular surface lipid accumulation on emerged silks of seven inbred lines; in that study, the relative abundance of fatty acids ranged from 10% to 19% of total extracellular lipids [19]. Observed differences in the abundances of different lipid classes (e.g. alkanes, alkenes, saturated and unsaturated fatty acids) can be attributed to differential accumulation patterns of individual extracellular surface lipid metabolites within each lipid class. As shown in S3 Fig, most metabolites in a given lipid class exhibit increased accumulation on emerged vs. husk-encased silks. For example, eight of the eleven alkane constituents observed on B73 silks harvested 3-days PSE showed 2- to 8-fold higher accumulation in emerged silks as compared to husk-encased silks (Panel A in S3 Fig). Therefore, increased accumulation of hydrocarbons and fatty acids observed on emerged silks is not due to the differential accumulation of only a select number of individual metabolites, but instead due to increased accumulation across most metabolites within that lipid class.

Interestingly, the relative abundances of unsaturated metabolites (i.e. unsaturated fatty acids and the alkene class of hydrocarbons) differed significantly between genotypes, with a higher relative abundance of unsaturated metabolites in Mo17. For example, in husk-encased silks at both 3- and 6-days PSE, the Mo17 inbred accumulated a higher proportion (approx. 15% greater) of alkenes than B73 (p<0.01; Fig 7C and 7D), which can be attributed to increased accumulation of individual alkenes (p<0.05) with chain lengths greater than 25 carbons in inbred Mo17 as compared to B73 (e.g. C27:1(7), C29:1(7), C29:1(9), C31:1(7) and C31:1(9) alkenes, compare panels B and K in S3 Fig). In contrast, for emerged silks relative abundances of alkenes did not differ between B73 versus Mo17, and only the relative abundance of unsaturated fatty acids was higher in Mo17 as compared to B73 at both 3- and 6-days PSE (p<0.05).

In our application of a novel extraction method for non-polar and amphipathic surface lipids, we have demonstrated that silks harvested at different PSE times, from different genotypes and between emerged and husk-encased portions of the silks, differ in concentrations of specific surface lipid metabolites as well as in relative abundances of specific surface lipid classes. Furthermore, the observed differences in the surface lipid metabolome between emerged and husk-encased silks, and between the two inbreds, suggests a potential role of the environment (emerged vs. husk-encased status) on surface lipid accumulation that is mediated by genotype; these observed differences, when combined with transcriptomics data, may also provide insights into the dynamics of the metabolic networks responsible for the synthesis of saturated and unsaturated hydrocarbon metabolites from fatty acid precursors [20].

## Conclusions

The one-step surface lipid extraction method using hexanes:diethyl ether (90:10) effectively extracts non-polar (i.e., linear hydrocarbons) and amphipathic metabolites (i.e., fatty acids, alcohols, and aldehydes) from plant tissues. Extraction with the hexanes:diethyl ether (90:10) solvent likely provides a better representation of extracellular surface lipids as compared to the traditionally used chloroform solvent, particularly in mutants that impact extracellular surface lipid composition. The optimized method allows for changes in extraction time that are amenable to handling large sample sizes in large-scale experiments without impacting metabolite recovery, and possibly limiting the potential contamination of surface lipid extracts with metabolites from internal lipid pools. Moreover, application of the extraction method further revealed the dynamic nature of the surface lipid metabolome on maize silks. Based on the reported findings, the described extraction method may have broader applicability for large-scale studies of the roles of cuticular lipids in plant systems and their importance in protection against environmental stresses, as well as the dissection of genetic and metabolic networks that underlie cuticular lipid production.

## Supporting information

S1 Fig Silk appearance after extraction in chloroform vs. hexanes:diethyl ether (90:10). Emerged silks from maize inbred B73 were extracted for 0, 1, 2, 3 or 4 min in either chloroform or hexanes:diethyl ether (90:10). Discoloration of the chloroform-extracted silks is minor at 1 min of extraction and increases at successive time points. In addition, structural integrity of silks extracted in chloroform was reduced. Notably, little discoloration is observed in silks extracted with hexanes:diethyl ether (90:10).
(TIF)

S2 Fig Representative gas chromatograms of extracellular surface lipids from wildtype and *gl1* seedling leaves extracted with either chloroform or hexanes:diethyl ether (90:10) solvents. A. Metabolite profile of a representative *glossy1* seedling extracted with chloroform. B. Metabolite profile of a representative wildtype seedling extracted with chloroform. C. Metabolite profile of a representative *glossy1* seedling extracted with hexanes:diethyl ether (90:10). D. Metabolite profile of a representative wildtype seedling extracted with hexanes:diethyl ether (90:10). Components of interest are identified by carbon chain length (e.g. C20) and are color-coded based on lipid class. Internal standards are labeled as IS.
(TIF)

S3 Fig Evaluation of individual surface lipid constituents from B73 and Mo17 silks harvested 3- and 6-days post-silk emergence. Panels A-R: Individual metabolite abundances for husk-encased vs. emerged silks. For each genotype (B73 or Mo17) at each harvest point (3-days or 6-days PSE), a set of graphs are presented to depict abundances of metabolites per lipid class (saturated hydrocarbons, unsaturated hydrocarbons, saturated fatty acids, unsaturated fatty acids, aldehydes). Panels A-D: B73 silks harvested 3-days PSE; Panels E-H: B73 silks harvested 6-days PSE; Panels J-M: Mo17 silks harvested 3-days PSE; Panels N-R: Mo17 silks harvested 6-days PSE. Panels A, E, J and N: saturated hydrocarbons; B, F, K, O: unsaturated hydrocarbons; C, G, L, P: saturated fatty acids; D, H, M, Q: unsaturated fatty acids; I, R: aldehydes. Asterisks denote statistically significant differences in accumulation between husk-encased and emerged silks, at p<0.05 according to a Tukey’s HSD test.
(XLSX)

S1 Table Concentrations of lipid metabolites extracted from fresh B73 silks. (XLSX)

S2 Table Concentrations of lipid metabolites extracted from lyophilized B73 silks. (XLSX)

S3 Table Concentrations of lipid metabolites extracted from B73 silks using different solvents and different extraction times. (XLSX)

S4 Table Concentrations of lipid metabolites extracted from seedling leaves from B73 plants. (XLSX)

S5 Table Concentrations of lipid metabolites extracted from seedling leaves of wildtype and *glossy1* plants. (XLSX)

S6 Table Concentrations of lipid metabolites extracted from silks from B73 and Mo17 plants. (XLSX)
