# Supplementary figures and images for: A robust and efficient method for the extraction of plant extracellular surface lipids as applied to the analysis of silks and seedling leaves of maize
Source: PLoS One. 2017 Jul 11;12(7):e0180850. doi: 10.1371/journal.pone.0180850 (PMC5507477; doi:10.1371/journal.pone.0180850)

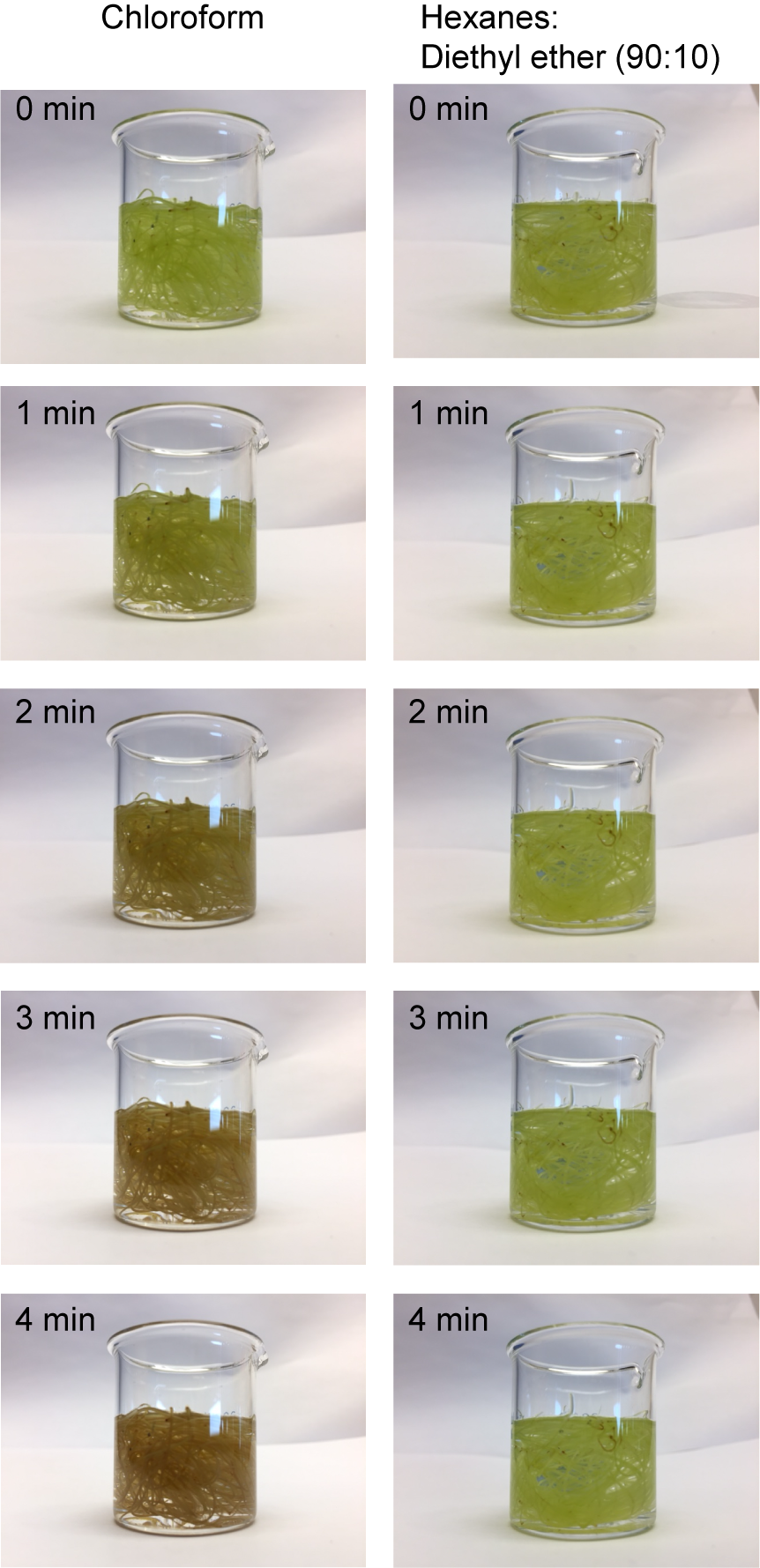

Supplement: S1 Fig — Emerged silks from maize inbred B73 were extracted for 0, 1, 2, 3 or 4 min in either chloroform or hexanes:diethyl ether (90:10). Discoloration of the chloroform-extracted silks is minor at 1 min of extraction and increases at successive time points. In addition, structural integrity of silks extracted in chloroform was reduced. Notably, little discoloration is observed in silks extracted with hexanes:diethyl ether (90:10). (TIF) [file pone.0180850.s001.tif]

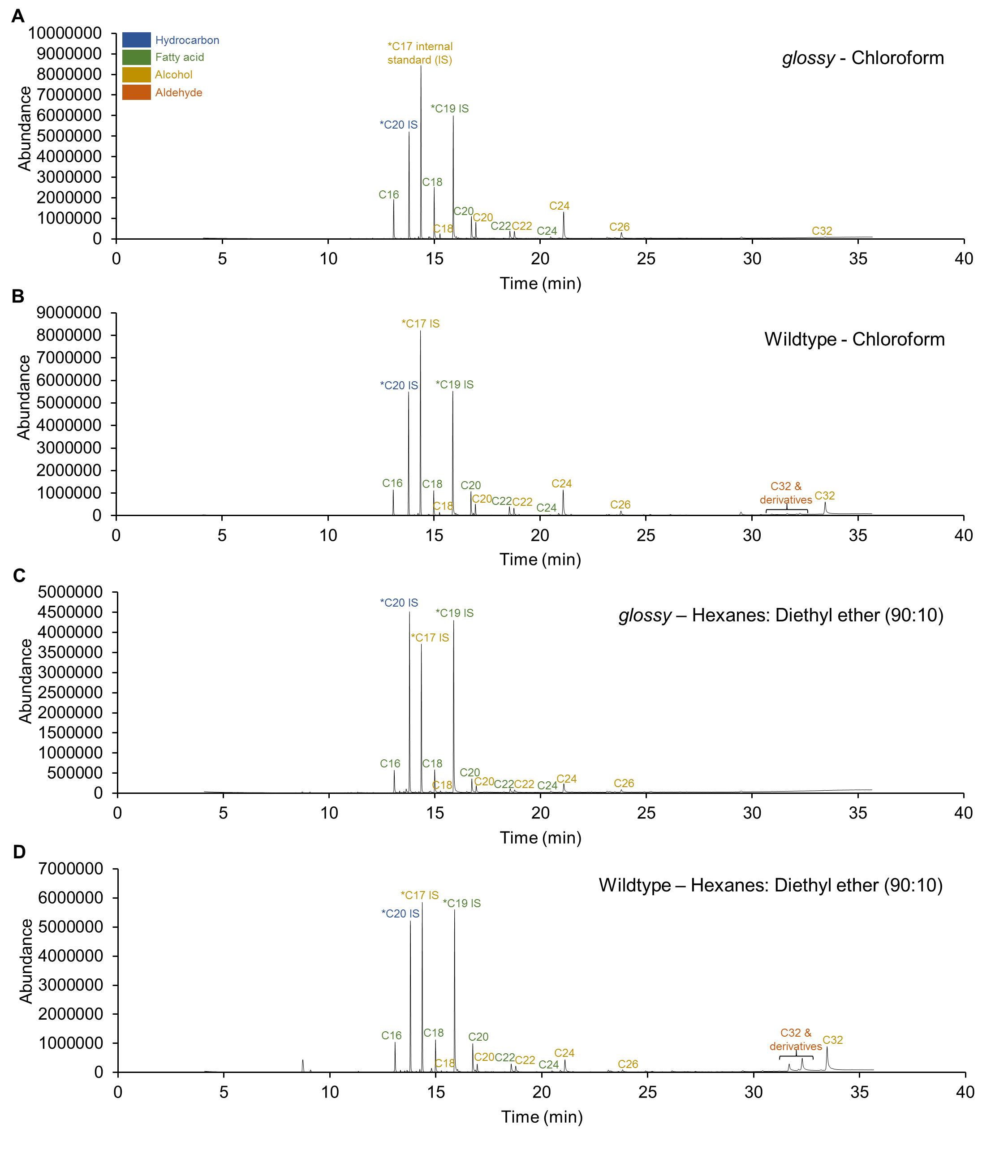

Supplement: S2 Fig — A. Metabolite profile of a representative glossy1 seedling extracted with chloroform. B. Metabolite profile of a representative wildtype seedling extracted with chloroform. C. Metabolite profile of a representative glossy1 seedling extracted with hexanes:diethyl ether (90:10). D. Metabolite profile of a representative wildtype seedling extracted with hexanes:diethyl ether (90:10). Components of interest are identified by carbon chain length (e.g. C20) and are color-coded based on lipid class. Internal standards are labeled as IS. (TIF) [file pone.0180850.s002.tif]
